# Frequent somatic transfer of mitochondrial DNA into the nuclear genome of human cancer cells

**DOI:** 10.1101/gr.190470.115

**Published:** 2015-06

**Authors:** Young Seok Ju, Jose M.C. Tubio, William Mifsud, Beiyuan Fu, Helen R. Davies, Manasa Ramakrishna, Yilong Li, Lucy Yates, Gunes Gundem, Patrick S. Tarpey, Sam Behjati, Elli Papaemmanuil, Sancha Martin, Anthony Fullam, Moritz Gerstung, ICGC Prostate Cancer Working Group, ICGC Bone Cancer Working Group, ICGC Breast Cancer Working Group, Jyoti Nangalia, Anthony R. Green, Carlos Caldas, Åke Borg, Andrew Tutt, Ming Ta Michael Lee, Laura J. van't Veer, Benita K.T. Tan, Samuel Aparicio, Paul N. Span, John W.M. Martens, Stian Knappskog, Anne Vincent-Salomon, Anne-Lise Børresen-Dale, Jórunn Erla Eyfjörd, Adrienne M. Flanagan, Christopher Foster, David E. Neal, Colin Cooper, Rosalind Eeles, Sunil R. Lakhani, Christine Desmedt, Gilles Thomas, Andrea L. Richardson, Colin A. Purdie, Alastair M. Thompson, Ultan McDermott, Fengtang Yang, Serena Nik-Zainal, Peter J. Campbell, Michael R. Stratton

**Affiliations:** 1 Cancer Genome Project, Wellcome Trust Sanger Institute, Hinxton, Cambridge CB10 1SA, United Kingdom;; 2 Cytogenetics Facility, Wellcome Trust Sanger Institute, Hinxton, Cambridge CB10 1SA, United Kingdom;; 3 Cambridge University Hospitals NHS Foundation Trust, Cambridge CB2 0QQ, United Kingdom;; 4 Department of Haematology, University of Cambridge, Cambridge CB2 0XY, United Kingdom;; 5 Cancer Research UK (CRUK) Cambridge Institute, University of Cambridge, Cambridge CB2 0RE, United Kingdom;; 6 BioCare, Strategic Cancer Research Program, SE-223 81 Lund, Sweden;; 7 CREATE Health, Strategic Centre for Translational Cancer Research, SE-221 00 Lund, Sweden;; 8 Department of Oncology and Pathology, Lund University Cancer Center, SE-221 85 Lund, Sweden;; 9 Breakthrough Breast Cancer Research Unit, Research Oncology, King's College London, Guy's Hospital, London SE1 9RT, United Kingdom;; 10 Laboratory for International Alliance on Genomic Research, RIKEN Center for Integrative Medical Sciences, 230-0045 Yokohama, Japan;; 11 National Center for Genome Medicine, Institute of Biomedical Sciences, Academia Sinica, Taipei 115, Taiwan;; 12 Department of Laboratory Medicine, Helen Diller Family Comprehensive Cancer Center, University of California, San Francisco, California 94158, USA;; 13 Netherlands Cancer Institute, 1066 CX Amsterdam, Netherlands;; 14 Department of General Surgery, Singapore General Hospital, Singapore 169608;; 15 Department of Molecular Oncology, British Columbia Cancer Agency, Vancouver V5Z 1L3, Canada;; 16 Department of Radiation Oncology and Department of Laboratory Medicine, Radboud University Medical Center, 6525 HP Nijmegen, Netherlands;; 17 Department of Medical Oncology, Erasmus MC Cancer Institute, Erasmus University Medical Center, 3015 CE Rotterdam, Netherlands;; 18 Section of Oncology, Department of Clinical Science, University of Bergen, N-5020 Bergen, Norway;; 19 Department of Oncology, Haukeland University Hospital, 5021 Bergen, Norway;; 20 Institut Curie, INSERM U934 and Department of Tumor Biology, 75248 Paris cédex 05, France;; 21 Department of Genetics, Institute for Cancer Research, Oslo University Hospital, The Norwegian Radium Hospital, Montebello, 0310 Oslo, Norway;; 22 The K.G. Jebsen Center for Breast Cancer Research, Institute for Clinical Medicine, Faculty of Medicine, University of Oslo, 0450 Oslo, Norway;; 23 Cancer Research Laboratory, University of Iceland, 101 Reykjavik, Iceland;; 24 Royal National Orthopaedic Hospital, Middlesex HA7 4LP, United Kingdom;; 25 UCL Cancer Institute, University College London, London WC1E 6DD, United Kingdom;; 26 University of Liverpool and HCA Pathology Laboratories, London WC1E 6JA, United Kingdom;; 27 Urological Research Laboratory, Cancer Research UK Cambridge Research Institute, Cambridge CB2 0RE, United Kingdom;; 28 Department of Surgical Oncology, University of Cambridge, Addenbrooke's Hospital, Cambridge CB2 0QQ, United Kingdom;; 29 Institute of Cancer Research, Sutton, London SM2 5NG, United Kingdom;; 30 Department of Biological Sciences and School of Medicine, University of East Anglia, Norwich NR4 7TJ, United Kingdom;; 31 Division of Genetics and Epidemiology, The Institute of Cancer Research, Sutton SM2 5NG, United Kingdom;; 32 Royal Marsden NHS Foundation Trust, London SW3 6JJ and Sutton SM2 5PT, United Kingdom;; 33 University of Queensland, School of Medicine, Brisbane, QLD 4006, Australia;; 34 Pathology Queensland, Royal Brisbane and Women's Hospital, Brisbane, QLD 4029, Australia;; 35 University of Queensland, UQ Centre for Clinical Research, Brisbane, QLD 4029, Australia;; 36 Breast Cancer Translational Research Laboratory, Université Libre de Bruxelles, Institut Jules Bordet, 1000 Brussels, Belgium;; 37 Université Lyon 1, Institut National du Cancer (INCa)–Synergie, 69008 Lyon, France;; 38 Dana-Farber Cancer Institute, Boston, Massachusetts 02215, USA;; 39 Brigham and Women's Hospital, Harvard Medical School, Boston, Massachusetts 02115, USA;; 40 Department of Pathology, Ninewells Hospital and Medical School, Dundee DD1 9SY, United Kingdom;; 41 Department of Surgical Oncology, University of Texas MD Anderson Cancer Center, Houston, Texas 77030, USA

## Abstract

Mitochondrial genomes are separated from the nuclear genome for most of the cell cycle by the nuclear double membrane, intervening cytoplasm, and the mitochondrial double membrane. Despite these physical barriers, we show that somatically acquired mitochondrial-nuclear genome fusion sequences are present in cancer cells. Most occur in conjunction with intranuclear genomic rearrangements, and the features of the fusion fragments indicate that nonhomologous end joining and/or replication-dependent DNA double-strand break repair are the dominant mechanisms involved. Remarkably, mitochondrial-nuclear genome fusions occur at a similar rate per base pair of DNA as interchromosomal nuclear rearrangements, indicating the presence of a high frequency of contact between mitochondrial and nuclear DNA in some somatic cells. Transmission of mitochondrial DNA to the nuclear genome occurs in neoplastically transformed cells, but we do not exclude the possibility that some mitochondrial-nuclear DNA fusions observed in cancer occurred years earlier in normal somatic cells.

Somatically acquired structural rearrangements are common features of the nuclear genomes of cancer cells. These may range from simple chromosomal rearrangements (Campbell et al. 2008) to more complex, compound patterns, such as chromothripsis (Stephens et al. 2011) and chromoplexy (Baca et al. 2013), or mobilization of transposable elements (Lee et al. 2012; Tubio et al. 2014). Intrachromosomal rearrangements are generally more common than interchromosomal rearrangements, indicating a higher likelihood of joining a double-strand break in a chromosome to another break in the same chromosome despite the availability of a much larger quantity of nuclear DNA from other chromosomes (Stephens et al. 2009).

In addition to the nuclear genome, human cells have a few hundred to a few thousand mitochondria, each carrying one or a few copies of the 16,569-bp-long circular mtDNA (Smeitink et al. 2001; Friedman and Nunnari 2014; Ju et al. 2014). During endosymbiotic co-evolution, most of the genetic information present in the ancestral mitochondrion has transferred to the nuclear genome (Gray et al. 1999; Adams and Palmer 2003; Timmis et al. 2004). An apparent burst of mtDNA transfer occurred during primate evolution ∼54 million years ago (Gherman et al. 2007) and occasional, probably more recent, transfer in humans has been observed in the germline (Turner et al. 2003; Goldin et al. 2004; Chen et al. 2005; Millar et al. 2010; Dayama et al. 2014). Although mtDNA nuclear transfer in a HeLa cell line derivative, and thus occurring in vitro, has been reported (Shay et al. 1991), de novo nuclear transfer of mtDNA in animal somatic tissues has not previously been comprehensively studied to our knowledge. To investigate the possibility of somatic mitochondrial-nuclear DNA fusion, we analyzed next-generation paired-end DNA whole-genome sequencing data from 559 primary cancers, 28 cancer cell lines (referred as 587 cancer whole genome below) and normal DNAs from the same individuals (Supplemental Table 1).

## Results

### Discovery of somatic mtDNA transfers to cancer nuclear genomes

**Figure 1.**
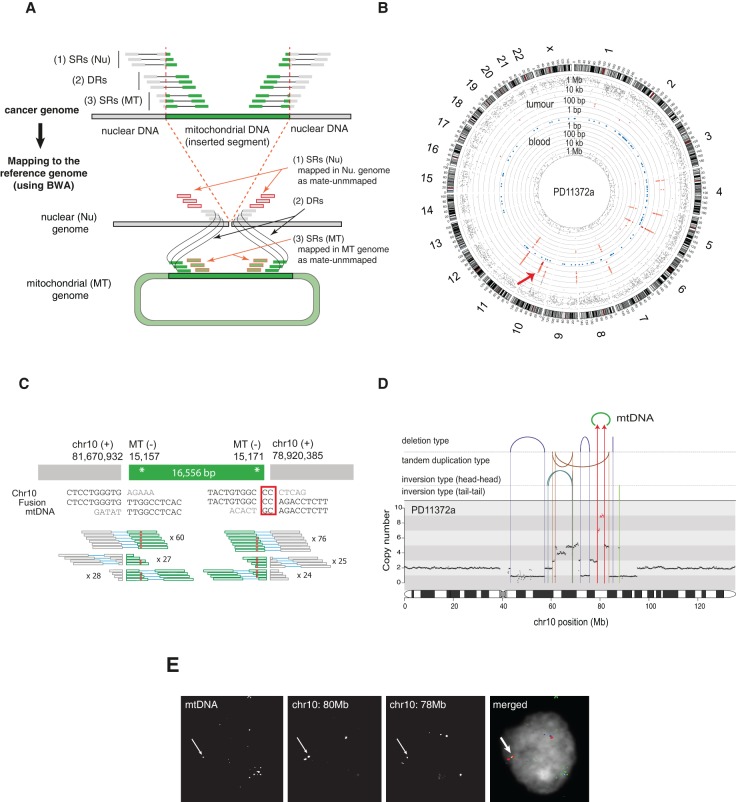
Discovery of somatic nuclear mtDNA transfer from PD11372a. (*A*) The strategy for detection of nuclear mtDNA transfer events. See Methods for a detailed description. (SRs) Split-reads, (DRs) discordant reads, (Nu) nucleus, (MT) mitochondria. (*B*) Graphical representation of discordant read clusters in PD11372a and its paired-normal tissue (PD11372b). The red arrow indicates tumor-specific discordant-read clusters in Chr 10. Chromosome ideograms are shown in the *outer* layer. The distance between each discordant read and one prior to it (the inter-read distance) is plotted on the vertical axis on a log-scale in the *middle* (tumor) and *inner* layer (blood). Blue dots shown in the *middle* layer represent known numts. (*C*) mtDNA integration in PD11372a. Breakpoint sequences are shown. Red rectangle highlights microhomology. Numbers of discordant split reads are presented. Inherited mtDNA substitution polymorphisms are shown by red asterisks. (*D*) Rearrangement architectures of Chromosome 10 of PD11372a. DNA copy numbers are shown by black dots. The copy number for 2.75-Mb-long region fused with mtDNA is colored in red. Reads supporting rearrangements (large deletions, tandem duplications, tail-tail and head-head inversions) are shown by arcs and vertical lines. Chr 10-mtDNA fusions are shown with red arrows. (*E*) Nuclear FISH confirms the mitochondrial-nuclear DNA fusion in the nucleus. (Red) Chr 10 (80 Mb), (blue) Chr 10 (78 Mb), and (green) mtDNA.

From the 587 pairs of cancer and normal whole-genome sequencing data, we searched for cancer-specific clusters of discordant paired-end sequence reads in which one member of the read-pair mapped to the nuclear genome and the other to the mitochondrial genome, and then characterized the nuclear-mitochondrial genome junctions to nucleotide resolution using individual sequence reads that bridged the junction (Fig. 1A). In 12 samples (overall positive rate 2.0%, 12 out of 587 samples), we observed 25 cancer-specific mitochondrial-nuclear DNA junctions (Table 1; Supplemental Figs. 1–6). Given that there are two junctions for a single integration event, we conclude that there are most likely 16 independent mtDNA insertions (Table 1). In addition to somatic transfers, we observed several novel rare germline (inherited) events that were shared between cancer and paired normal samples (Supplemental Table 2; Supplemental Material).

**Table 1.**
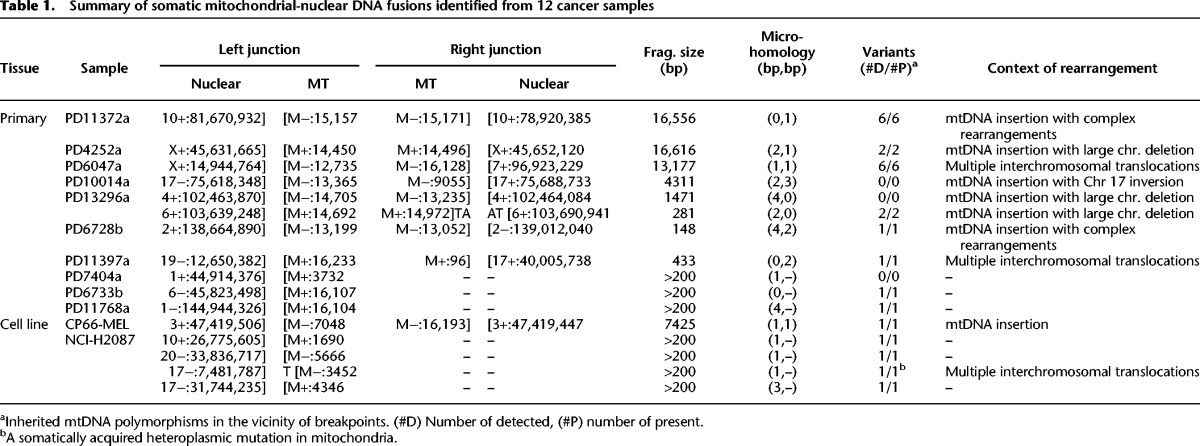
Summary of somatic mitochondrial-nuclear DNA fusions identified from 12 cancer samples

Breast cancer PD11372a showed a somatically acquired integration of almost the entire human mtDNA sequence (16,556 bp) into a highly amplified 2.75-Mb-long region of Chromosome 10q22.3. The integration event was strongly supported by both discordant and split read clusters (Fig. 1B–D) and was confirmed by short- and long-range PCR across the nuclear-mitochondrial genome junctions (Supplemental Figs. 7, 8; Supplemental Table 3). It was not found in normal tissue (blood) from the same individual or from all the other cases and did not match any known inherited nuclear mtDNA-like sequences (known as numts) (Gherman et al. 2007; Hazkani-Covo et al. 2010). Consistent with its somatic origin, the mtDNA fused to the nuclear genome harbored sequence polymorphisms identical to those present in the mitochondria of this individual (14,905 G > A; 15,028 C > A; 15,043 G > A; 15,326 A > G; 15,452 C > A, and 15,607 A > G). Fluorescence in situ hybridization (FISH) experiments performed on formalin-fixed paraffin embedded tissue confirmed that the fused DNA segment exists in the nuclei of cancer cells (Fig. 1E).

**Figure 2.**
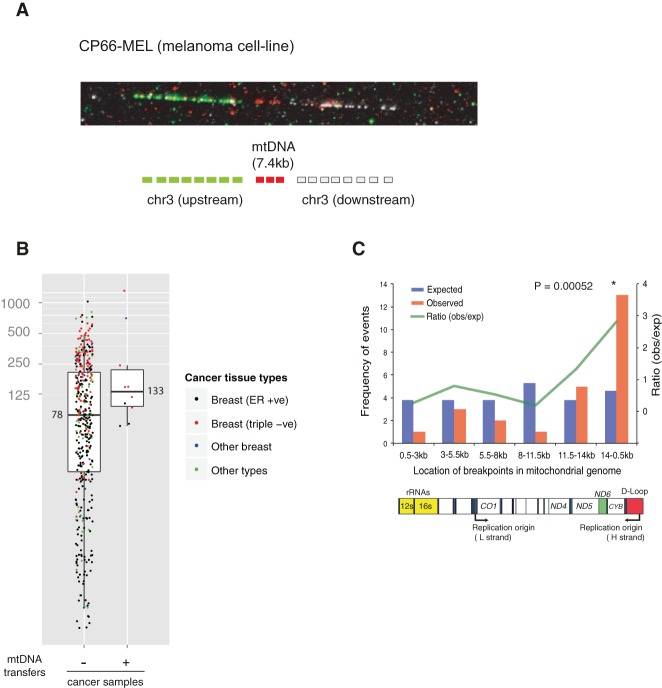
Features of somatic mtDNA nuclear transfer in 12 cancer samples. (*A*) Fiber FISH visualizes the mitochondrial-nuclear DNA fusion from the CP66-MEL cell line. (*B*) Positive correlation between mtDNA transfer and numbers of nuclear chromosomal rearrangements (large deletion, tandem duplication, inversion, and translocation) in cancer genomes. Median values are shown. (*C*) mtDNA breakpoints are enriched in the 14 kb- to 500-bp region of the MT genome. (*Top*) Blue and red bars represent the expected and observed numbers of breakpoints in each interval of MT genome, respectively. Green line shows ratio between observed and expected numbers. A χ^2^ test was applied to test enrichment. (*Bottom*) Schematic structural features of the MT genome corresponding to the intervals are shown.

In total, we found 10 primary cancers (1.8%, 10/559) and two cancer cell lines (7.1%, 2/28) with somatic mtDNA integrations into their nuclear genomes (Table 1; Supplemental Figs. 1–6). Of the 12 cancers, two (primary cancer PD13296a and cancer cell line NCI-H2087) had more than one mitochondrial-nuclear DNA translocation event. All integrations were supported by both discordant and split reads and further confirmed by PCR across the nuclear-mitochondrial genome junctions (Supplemental Fig. 7; Supplemental Table 3). All inherited mtDNA substitution polymorphisms near these breakpoints were detected (Table 1). To further visualize the transfer events, we performed high-resolution FISH on stretched DNA fibers (fiber FISH) from the melanoma cell line, CP66-MEL (Fig. 2A).

### Somatic nuclear integration of mtDNA is frequently combined with other rearrangements of the nuclear genome

The rate of somatic nuclear transfer of mtDNA may vary according to tumor type. Triple-negative breast cancer showed a fivefold higher frequency compared to estrogen-receptor (ER) positive breast cancers (6.2% and 1.2%, respectively; Fisher's exact test *P* = 0.002). Triple-negative breast cancer genomes carry a higher number of chromosomal rearrangements than ER-positive breast (average 254 and 94, respectively, in our data set). As a result, there was a suggestive positive correlation between the number of chromosomal rearrangements and mtDNA transfers (Mann-Whitney *U* test, one-sided *P* = 0.05) (Fig. 2B).

The length of mtDNA fragments transferred ranged from 148 bp to entire mitochondrial genomes (16.5 kb) (Table 1). Interestingly, breakpoints in mtDNA were enriched near the mitochondrial genome heavy strand origin of replication (χ^2^ test, *P* = 0.0005) (Fig. 2C). This suggests that the generation of mtDNA segments to be integrated into the nuclear genome is not random and may occur in a mtDNA replication-dependent manner (Lenglez et al. 2010).

**Figure 3.**
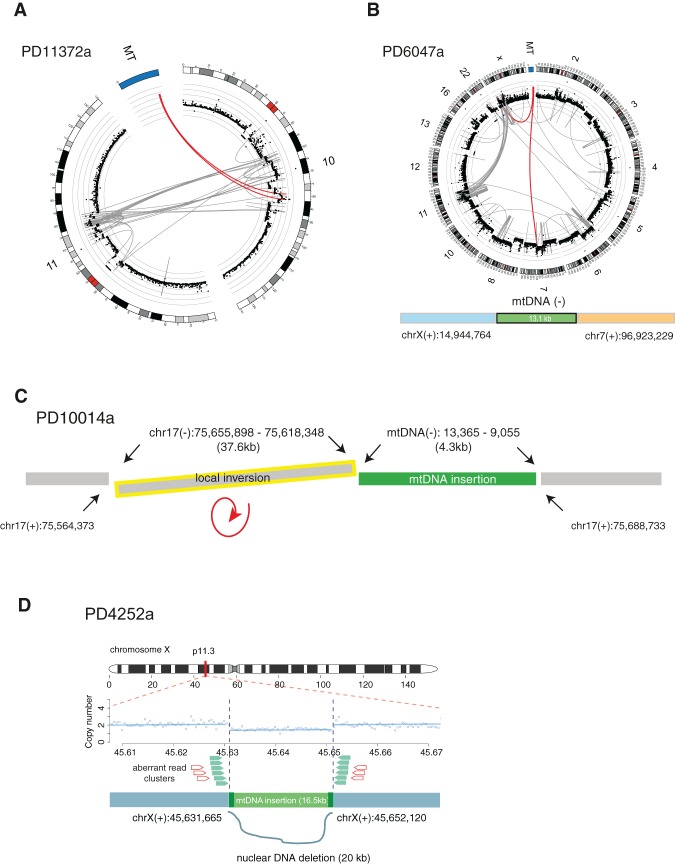
Concurrence of somatic mtDNA nuclear transfers with other structural variations. The complex web of rearrangements in the vicinity of mitochondrial-nuclear DNA fusions from four examples. (*A*) In PD11372a, mtDNA integration with complex rearrangements between Chr 10 and 11. (*B*) In PD6047a, mtDNA integration with complex rearrangements among Chr 6, 7, 11, 22, and X. (*A*,*B*) DNA copy numbers are shown by black dots with a log scale. Red lines represent translocations involving mtDNA. (*C*) In PD10014a, mtDNA integration combined with a local inversion (yellow). (*D*) In PD4252a, mtDNA integration with a local deletion. DNA copy numbers are shown with blue dots and lines. Aberrant read clusters (discordant and split reads) are shown by green and red arrows, respectively.

Of the 25 mitochondrial-nuclear DNA junctions, at least 17 (68.0%) were clearly associated with other nuclear chromosomal rearrangements (e.g., inversions, translocations, and large deletions) in the vicinity (Table 1; Supplemental Figs. 1–6). For example, with respect to PD11372a described earlier, genomic fragments from Chromosomes 10, 11, and mtDNA generated complex derivative chromosomes (Fig. 3A). In PD6047a, an mtDNA fragment was involved in chains of complex genomic translocations involving Chromosomes 6, 7, 11, 22, and X (Fig. 3B). In PD10014a, a local inversion was combined with the mtDNA integration event (Fig. 3C), and in PD4252a, a 16.5-kb mtDNA integration was found in a position on the X Chromosome from which ∼20 kb of nuclear DNA had been somatically deleted (Fig. 3D). Thus, mtDNA is often integrated into nuclear genomes in the vicinity of, or as part of, complex rearrangements. Although germline numts tend to occur near transposable elements such as LINEs and SINEs (Mishmar et al. 2004), we do not observe this association for somatic events (χ^2^ test, two-sided *P* = 0.33) (Supplemental Table 4).

### The mechanism and timing of somatic nuclear transfer of mtDNA

**Figure 4.**
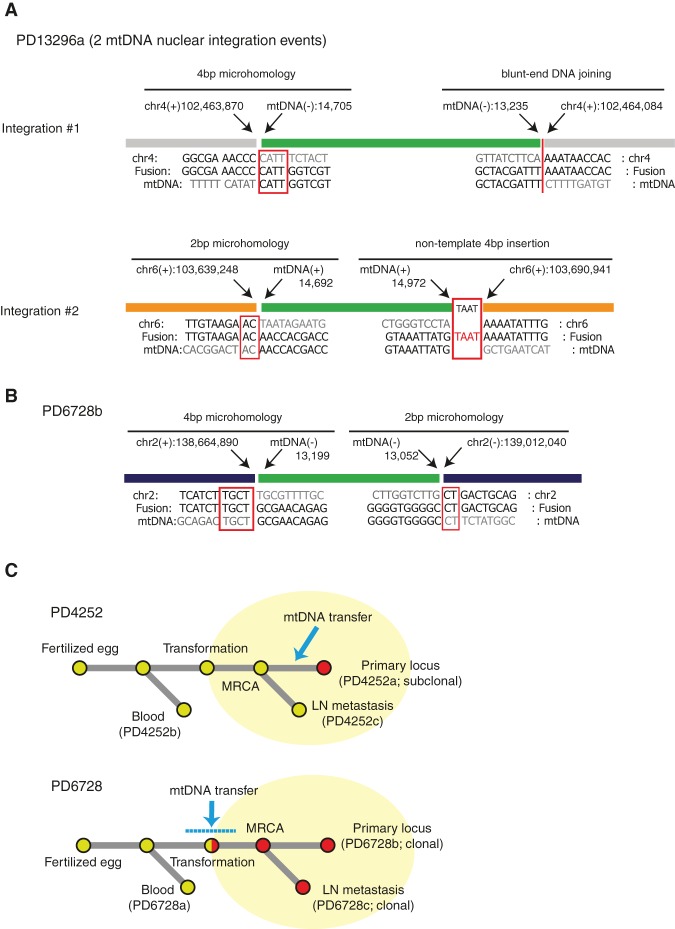
Nucleotide-resolution breakpoint sequences and the timing of somatic mtDNA nuclear integration. (*A*) Breakpoint sequences of nuclear-mtDNA fusions in PD13296a. Red rectangles highlight sequence microhomology and nontemplate nucleotides insertion. (*B*) Breakpoint sequences of nuclear-mtDNA fusions in PD6728b. Red rectangles highlight sequence microhomology. (*C*) Phylogenetic trees showing the timing of somatic mtDNA nuclear transfers in PD4252 and PD6728 samples. (MRCA) Most recent common ancestor cell.

There was overlapping sequence microhomology (from 1 to 4 bp) in 20/25 breakpoints (80%) (Fig. 4A,B; Table 1; Supplemental Figs. 1–6), substantially more than expected by chance (χ^2^ test, *P* = 5 × 10^−26^). Thus, DNA sequence microhomology plays an important role in mitochondrial-nuclear DNA integration events, although blunt-end DNA repair was also observed. In two breakpoints, we also found nontemplated short-nucleotide insertions (1 and 4 bp long) (Fig. 4A; Table 1). Overall, these features are characteristic of DNA double-strand break repair by nonhomologous end joining (NHEJ) (Hastings et al. 2009). However, they do not rule out replication-based mechanisms switching template between nuclear and mtDNA, such as microhomology-mediated break-induced replication (MMBIR) (Liu et al. 2011).

We investigated the timing of somatic mtDNA integration into the nuclear genome by assessing cases in which a metastatic sample had been sequenced in addition to the primary tumor. One such case (PD4252a) showed the mitochondrial-nuclear integration event in the primary but not in the metastasis (Fig. 4C), indicating that mtDNA transfer to the nucleus can occur after neoplastic transformation and during the course of subclonal evolution of the cancer. The other (PD6728b) showed it in both the primary and metastasis (Fig. 4C), suggesting that this event occurred in the common ancestral cancer clone or in normal somatic cells prior to neoplastic change.

### Nuclear transfer of mtDNA is unexpectedly frequent in human somatic cells

**Figure 5.**
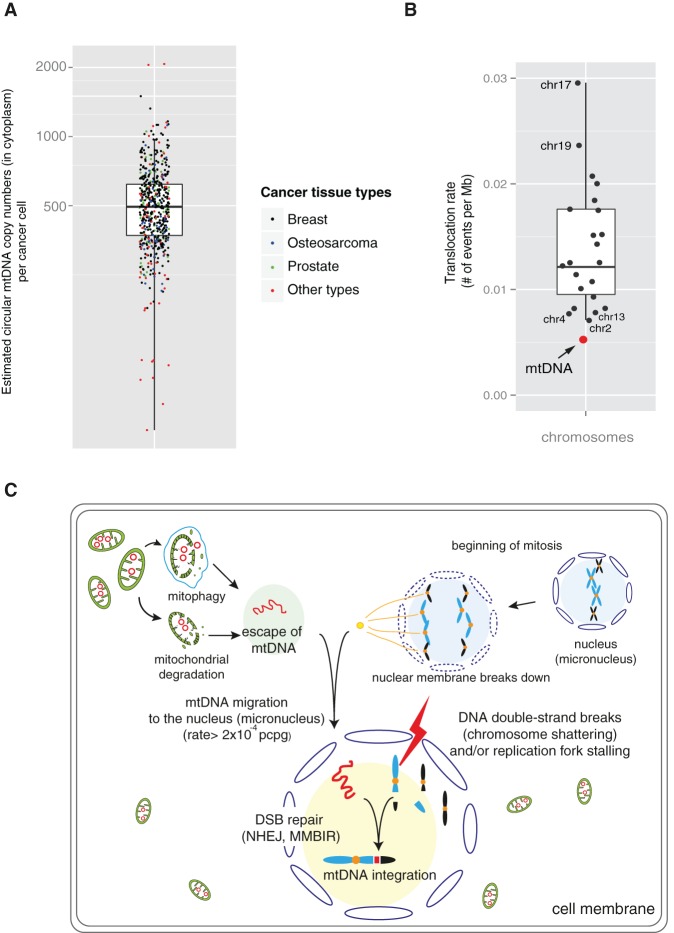
Frequency and potential mechanisms of somatic mtDNA nuclear transfer in human cancer. (*A*) Estimated circular mtDNA copy numbers (in the cytoplasm) per cancer cell from 587 cancer tissues sequenced. The ratio of read depths between autosomes and mtDNA was used (see Methods). (*B*) Similar frequency of somatic nuclear mtDNA integrations compared to the frequency between autosomes (chromosomal translocation). (*C*) A model of somatic mtDNA transfer to the nuclear genomes.

To obtain a perspective on the frequency of mitochondrial-nuclear DNA translocation, we compared its rate to that of intranuclear interchromosomal translocation, taking into account the sizes and copy numbers of the mitochondrial and nuclear genomes. Our sequencing data suggest that each cancer cell carries ∼500 copies of circular mtDNA (median value 495) (Fig. 5A), amounting in aggregate to ∼8 million base pairs (bp) of mtDNA (500 copies × 16.5 kb) enclosed by the mitochondrial double membrane in the cytoplasm of each cancer cell. The average frequency in the cancers analyzed of mitochondrial-nuclear DNA fusion was 5.1 × 10^−3^ junctions per million bp of mtDNA, only half the average rate of intranuclear interchromosomal translocation (1.2 × 10^−2^ junctions per million bp) and similar to that of Chromosomes 2, 4, and 13 (Fig. 5B). Given the multiple physical barriers to contact between the two genomes, the results indicate remarkably high rates of mtDNA escape, contact, and/or integration with nuclear DNA in human cancer cells. These appear to be considerably higher than in the germline across human evolution but comparable to those observed in *Saccharomyces cerevisiae* (Thorsness and Fox 1990) and for chloroplast DNA migration into the nucleus in tobacco plants (Methods; Supplemental Material; Huang et al. 2003).

## Discussion

Despite multiple physical barriers, there are plausible mechanisms by which mtDNA and nuclear DNA could come into contact (Fig. 5C). Free mtDNA can be released into the cytoplasm from degrading mitochondria or after mitophagy (Zhang et al. 2008; Eiyama et al. 2013; Higgins and Coughlan 2014). Degradation of mitochondria may be accelerated in cancer cells due to hypoxia and increased energy demands (Zhang et al. 2008; Eiyama et al. 2013; Higgins and Coughlan 2014). Even without a bespoke molecular process for transportation, mtDNA could then, in principle, migrate to the nucleus during mitotic metaphase or anaphase when the nuclear membrane has broken down. When these events are coupled with concurrent double-strand breaks (DSBs) and/or replication fork stalling of nuclear chromosomal DNA, mtDNA could be picked up and integrated into the nuclear genome as part of the process of rejoining DSBs (NHEJ) (Hastings et al. 2009) or used as an alternative DNA template in replication (MMBIR) (Liu et al. 2011). Micronuclei in cancer cells, which can be generated by errors in segregation of mitotic nuclear chromosomes, may contribute to the events. Chromosomes in micronuclei frequently undergo defective and delayed DNA replication, resulting in extensive fragmentation with subsequent jumbled rejoining compared to their original order and orientation (Crasta et al. 2012; Forment et al. 2012). Thus, mtDNA fragments incorporated into micronuclei could end up fused to shattered nuclear chromosomes. It is worthy of note that mtDNA escaping to the nucleus can be actively used for DNA repair in *Saccharomyces cerevisiae* (Ricchetti et al. 1999; Yu and Gabriel 1999), particularly when error-free DSB DNA repair is not possible. Whether this applies in mammalian cells is unknown.

Some of the somatic nuclear mtDNA integrations we identified are directly adjacent to nuclear genes. For example, nuclear-mtDNA fusion in PD11372a occurred in the fifth intron of the *KCNMA1* gene, a potassium channel frequently amplified in prostate and breast cancers (Oeggerli et al. 2012). However, we do not find obvious enrichment of the nuclear-mtDNA fusion breakpoints near human nuclear genes. RNA-seq from the NCI-H2087 cell-line indicates that mtDNA fragments in the nucleus of the cell line are not expressed as parts of mitochondrial-nuclear fusion transcripts. Thus, the majority of the nuclear mtDNA translocation events are likely to be passenger events, similar to mutations of all other types in most cancer genomes. However, we do not exclude the possibility that some of these events may have functional consequences in human cancer by generating fusion mRNA transcripts (Shay et al. 1991) and/or truncating cancer genes by mtDNA insertion within exons.

This study has shown that fusion of mtDNA to nuclear DNA occurs in human somatic cells at a rate similar to that of translocation between nuclear chromosomes. Physical migration of mtDNA into the nucleus may be much more frequent in stem cells than ones in a terminally differentiated stage (Schneider et al. 2014). Further studies will need to address the mechanisms by which the apparent physical barriers to contact between mitochondrial and nuclear DNA are so effectively overcome.

## Methods

### Samples and sequencing data

We analyzed 559 primary tumors and 28 cancer cell-lines in this study. Paired-normal samples for all the cancers were also included in this study in parallel. Whole-genome sequences used in this study were generated by Illumina platforms (either Genome Analyzer or HiSeq 2000). Cancer genomes were sequenced to at least 25× coverage. With respect to TCGA data, we downloaded aligned BAM files through UCSC CGHub (http://cghub.ucsc.edu). Sequencing reads were aligned on the human reference genome build 37 (GRCh37) and human reference mtDNA sequence (revised Cambridge reference sequence, rCRS) (Andrews et al. 1999), mainly by the BWA alignment tool (Li and Durbin 2009). SAMtools (Li et al. 2009) was used for manipulating sequence reads.

### Calling mitochondrial-nuclear DNA fusion events

We employed a pipeline for identification of putative mtDNA translocation to chromosomal DNA (Fig. 1A). From paired-end whole-genome sequencing data of tumors, we extracted discordant reads (DRs), where one end aligned uniquely to mtDNA and the other end to nuclear DNA. In all cases, both ends must have a mapping quality greater than zero. Those discordant reads are clustered together using the following criteria: reads sharing (1) close alignment positions (<500 nucleotides) for both ends on nuclear and mtDNA, and (2) the same orientations. In order to remove false positives, we removed clusters supported by less than five discordant reads.

In order to remove potential germline calls, several filters are applied to the tumor candidate cluster. The clusters from tumor cells were removed if they overlap with clusters identified from matched and/or unmatched normal tissues by more tolerable criteria (supported by more than one discordant read) from (1) its paired-normal tissue, and (2) from the other 586 unmatched normals. Filtered clusters were further refined with known germline human numts, a combined set from the human reference genome (hg19) detected by BLAT (Kent 2002) (*n* = 123) and from Simone et al. (2011) (*n* = 766). Finally, 25 clusters were selected as somatic candidates.

### Nucleotide-resolution breakpoints for the translocation junctions

To obtain nucleotide-resolution breakpoints, we searched for split-reads (SRs) with one of the ends spanning the junction of the translocation. We extracted “orphan” or “mate-unmapped” reads (one end of a read is unmapped by the BWA aligner) in the vicinity (<1000 bp) of discordant-read clusters on nuclear and mitochondrial genome sequences. Sequences from the unmapped end are then re-aligned by BLAT (Kent 2002), which enables split-read mapping.

### Validation by PCR

A PCR validation assay of the somatic mtDNA transfer was performed using genomic DNA from both cancer and paired-normal tissues. Primers were designed to amplify all the breakpoints (Supplemental Table 3). The short-fragment PCR reactions were performed as previously described (Tubio et al. 2014). With respect to long-range PCR, elongation time was increased 1 min per 1 kb.

### Generation of FISH probes

Human bacterial artificial chromosomes (BAC) and fosmid clones used in this study were obtained from the clone archive team of the Wellcome Trust Sanger Institute. Plasmid DNA was prepared using the PhasePrep BAC DNA kit (Sigma-Aldrich). Human mtDNA was isolated from lymphoblastoid cells using a Mitochondrial DNA Isolation kit (Abcam).

Probes for use in FISH were made as described before (Gribble et al. 2013). Purified mtDNA and plasmid DNA were first amplified using a GenomePlex Whole Genome Amplification (WGA) kit (Sigma-Aldrich) following the manufacturer's protocols, then labeled using a WGA reamplification kit (Sigma-Aldrich) with a custom-made dNTP mix. Probes for interphase FISH were labeled directly with Aminoallyl-dUTPs - ATTO-488, -Cy3, -Texas Red, and -Cy5 (Jena Bioscience); probes for fiber-FISH were labeled with Biotin-16-dUTP, Digoxigenin-11-dUTP (Roche), and DNP-11-dUTP (PerkinElmer).

### Validation by fiber-FISH with single-molecule DNA fibers generated by molecular combing

Single-molecule DNA fibers from the cancer cell line, CP66-MEL, were prepared by molecular combing (Michalet et al. 1997) following the manufacturer's instructions (Genomic Vision). Briefly, the cells were embedded in a low-melt-point agarose plug (1 million cells per plug), followed by proteinase K digestion, washing in 1 × TE (10 mM Tris, 1 mM EDTA, pH 8.0) and beta-agarose digestion steps. The DNA fibers were mechanically stretched onto saline-coated coverslips using a Molecular Combing System (Genomic Vision).

For fiber-FISH, ∼500 ng of labeled DNA from each probe and 4 μg of human Cot-1 DNA (Invitrogen) were precipitated using ethanol, then resuspended in a mix (1:1) of hybridization buffer (containing 2 × SSC, 10% sarkosyl, 2 M NaCl, 10% SDS, and blocking aid [Invitrogen]) and deionized formamide (final concentration 50%). Coverslips coated with combed DNA fibers were dehydrated through a 70%, 90%, and 100% ethanol series and aged at 65°C for 30 sec, followed by denaturation in an alkaline denature solution (0.5 M NaOH, 1.5 M NaCl) for 1–3 min, three washes with 1×PBS (Invitrogen), and dehydration through a 70%, 90%, and 100% ethanol series. The probe mix was denatured at 65°C for 10 min before being applied onto the coverslips, and the hybridization was carried out in a 37°C incubator overnight. The post-hybridization washes consisted of two rounds of washes in 50% formamide/2 × SSC (v/v), followed by two additional washes in 2 × SSC. All post-hybridization washes were done at 25°C, 5 min each time. Digoxigenin-11-dUTP (Roche) labeled probes were detected using a 1:100 dilution of monoclonal mouse anti-dig antibody (Sigma-Aldrich) and a 1:100 of Texas Red-X-conjugated goat anti-mouse IgG (Molecular Probes/Invitrogen); DNP-11-dUTP (PerkinElmer) labeled probes were detected using a 1:100 dilution of Alexa 488-conjugated rabbit anti-DNP IgG and 1:100 Alexa 488-conjugated donkey anti-rabbit IgG (Molecular Probes/Invitrogen); biotin-16-dUTP (Roche) labeled probes were detected with one layer 1:100 of Cy3-avidin (Sigma-Aldrich). After detection, slides were mounted with SlowFade Gold mounting solution containing 4′,6-diamidino-2-phenylindole (Molecular Probes/Invitrogen). Images were visualized on a Zeiss AxioImager D1 microscope. Digital image capture and processing were carried out using the SmartCapture software (Digital Scientific UK).

### Nuclear interphase FISH

Nuclei extraction from paraffin-embedded tissue of patient PD11372a and interphase-FISH followed Paternoster et al. (2002), with the exception that 60-μm-thick sections were used in our study. The post-hybridization washes consisted of two rounds of washes in 50% formamide/2 × SSC (v/v), followed by two additional washes in 2 × SSC. Slides were mounted with SlowFade Gold mounting solution containing 4′,6-diamidino-2-phenylindole (Molecular Probes/Invitrogen). Images were captured and processed as described above.

### Correlation between somatic mtDNA integration site and transposable elements

We performed a study similar to the previous report (Mishmar et al. 2004). We calculated the distance between each mtDNA-insertion site (breakpoint) and its nearest transposable elements (either of SINE, LINE, LTR, simple repeat, or DNA transposon by RepeatMasker, downloaded from the UCSC Genome Browser, June 6, 2013). Then, each mtDNA-insertion site was categorized into one of four groups: (A) breakpoint within a transposable element; (B) breakpoint within 15 bp from a transposable element; (C) within 15–150 bp; and (D), >150 bp. In order to understand the positional enrichment of breakpoints from transposable elements, we randomly generated in silico breakpoint positions 40 times as many (total *n* = 1000) as we observed from each chromosome in the real data set. In silico breakpoints located within gaps of the human reference genome were removed and replaced by newly generated insertions. For these in silico-generated breakpoints, the distances from the nearest transposable elements were calculated and then categorized into one of the four groups (A,B,C, and D). Finally, the difference in the frequency of breakpoints in each group between the observed and in silico-generated data set was compared using a χ^2^ test.

### Assessment of mtDNA copy numbers

To understand mtDNA copy numbers in a cancer cell, we compared average read depth of coverage between 22 autosomes and mtDNA. With respect to the tumor sequences by whole-genome sequencing, average haploid autosomal coverage (RD_autosome_) was obtained from the read depth of 2.685-Gb-long autosomal regions (excluding chromosomal gaps). Likewise, average mtDNA coverage (RD_mtDNA_) was obtained from the read depth of the 16.5-kb mitochondrial genome. Finally, mtDNA copy number in a diploid cell (*C*_mt_) is calculated as shown below:

$$C_{\mathrm{mt}}=2\times \frac{{\mathrm{RD}}_{\mathrm{mtDNA}}}{{\mathrm{RD}}_{\mathrm{autosome}}}.$$

### Assessment of translocation rate for autosomes and mitochondria

We identified structural variations among nuclear chromosomes (large deletions, tandem duplications, inversions, and interchromosomal translocations) using the BRASS II algorithm (Nik-Zainal et al. 2012), which identifies rearrangements by clustering discordant read pairs that point to the same junction and confirms breakpoints by local assembly of unmapped reads. The sensitivity and specificity of the BRASS II algorithm is equivalent to those values of the algorithm used for mitochondrial-nuclear DNA fusions (data not shown). We extracted interchromosomal translocations to calculate the rate of such events. The rate of each haploid autosome (Rtr,ch) is calculated as shown below:

Rtr,ch(events per megabase)=Ntr,ch/(2×Lch)/Nsam,

where Ntr,ch is the total number of somatic interchromosomal translocation junctions involving a specific chromosome, Lch is the length of the nonredundant region of the chromosome in megabases, and Nsam is the total number of samples analyzed. To obtain the unique region length (Lch), we excluded redundant (or highly repetitive) sequence lengths from the ungapped length of each chromosome. Genomic regions classified in one or more of the three criteria shown below were defined as redundant, where translocation events could not be easily detected due to ambiguous read alignment: (1) simple repeats, located by Tandem Repeats Finder (Benson 1999); (2) segmental duplications with moderate to high sequence similarity (≥95%) (Bailey et al. 2002), or (3) repetitive sequences including up to 10 different classes of repeats (such as SINE, LINE, LTR, DNA transposons, and microsatellites), located by the RepeatMasker program (http://www.repeatmasker.org), with a low divergence level (divergence < 5%). These nonredundant sequence regions were downloaded from the UCSC Genome Browser (http://genome.ucsc.edu).

Similarly, the rate of mitochondrial-nuclear DNA translocations (Rtr,mt) was calculated as below:

$$\mathrm{Rtr},\mathrm{mt}(\text{events per megabase})=\mathrm{Ntr},\mathrm{mt}/(C_{\mathrm{mt}}\times L_{\mathrm{mtDNA}})/\mathrm{Nsam},$$

where Ntr,mt is the total number of junctions of somatic mitochondrial-nuclear DNA fusions identified, *C*_mt_ is the median value of mitochondrial genome copy numbers in a diploid cancer cell calculated above (495 copies), and *L*_mtDNA_ is the length of the mitochondrial genome in megabases (0.016569 Mb).

### Assessment of the rates of nuclear mtDNA fusion and mtDNA escape to the nucleus

Fusion of mtDNA to the nuclear genome requires at least two events, each of which could influence the rate of mitochondrial-nuclear DNA fusion. These include escape of mtDNA to the nucleus and integration to nuclear DNA. According to this model, the overall number of such fusion events can be calculated using the rates for these processes (*ρ*_escape_ and *ρ*_integration_, respectively):

$$\mathrm{Ntr}=\mathrm{Nsam}\times \mathrm{Ngen}\times \rho_{\mathrm{escape}}\times \rho_{\mathrm{integration}},$$

where Ntr is the number of total somatic mitochondrial-nuclear DNA fusion events (*n* = 12), Nsam is the total number of cancer tissues (*n* = 587), and Ngen is the number of average cell generation from the fertilized egg. Using a reasonable assumption that Ngen = 1000, we obtain the rate of somatic mtDNA fusion to the nuclear genome (*ρ*_escape_ × *ρ*_integration_) to be 2 × 10^−5^ per cell per cell generation (pcpg). With one more very conservative assumption that *ρ*_integration_ is 0.1, we obtain *ρ*_escape_ to be 2 × 10^−4^ pcpg, or at least one escape event per 5000 cell generations. We hypothesize that the real *ρ*_integration_ value is thought to be much lower than 0.1, which results in a higher *ρ*_escape_. For example, during the generation of knockout mice, homologous recombination allows one fixation event per 1000–10,000 microinjected DNA copies (Brinster et al. 1985). The integration rate may, however, be higher than the rate in cancer cells with defective homologous recombination-based repair and increased availability of nuclear double-strand breaks, which can be joined to by NHEJ or MMBIR.

The mtDNA fusion to the nuclear genome in the germline (the rate of numts insertion) is around 5 × 10^−6^ per germ cell per individual generation in previous phylogenetic studies (Hazkani-Covo et al. 2010). The rate is equivalent to ∼5 × 10^−8^ pcpg, given that the number of germ cell divisions per human generation is ∼100 (401 in males and 31 in females [Drost and Lee 1995]).

## Data access

Sequence data for sample pairs with positive mtDNA nuclear transfer have been submitted to the European Genome-phenome Archive (EGA; https://www.ebi.ac.uk/ega/home). The study accession number is EGAS00001001234. Sample accession numbers are available in Supplemental Table 1.

## Supplementary Material

Supplemental Material
