# Supplementary material for: Frequent somatic transfer of mitochondrial DNA into the nuclear genome of human cancer cells
Source: Genome Res. 2015 Jun;25(6):814–24. doi: 10.1101/gr.190470.115 (PMC4448678; doi:10.1101/gr.190470.115)
Supplement: Supplemental Material [file supp_25_6_814__index.html]

Frequent somatic transfer of mitochondrial DNA into the nuclear genome of human cancer cells — Frequent somatic transfer of mitochondrial DNA into the nuclear genome of human cancer cells — Supplemental Material 

# Frequent somatic transfer of mitochondrial DNA into the nuclear genome of human cancer cells

## Supplemental Material

**Files in this Data Supplement:**

- Supp Figure1.pdf
- Supp Figure2.pdf
- Supp Figure3.pdf
- Supp Figure4.pdf
- Supp Figure5.pdf
- Supp Figure6.pdf
- Supp Figure7.pdf
- Supp Figure8.pdf
- Supp Figure9.pdf
- Supplemental Material.docx
- TableS1.xlsx
- TableS2.xlsx
- TableS3.xlsx
- TableS4.xlsx
