# Supplementary material for: Frequent somatic transfer of mitochondrial DNA into the nuclear genome of human cancer cells
Source: Genome Res. 2015 Jun;25(6):814–24. doi: 10.1101/gr.190470.115 (PMC4448678; doi:10.1101/gr.190470.115)
Supplement: Supplemental Material [file supp_gr.190470.115_Supp_Figure3.pdf]

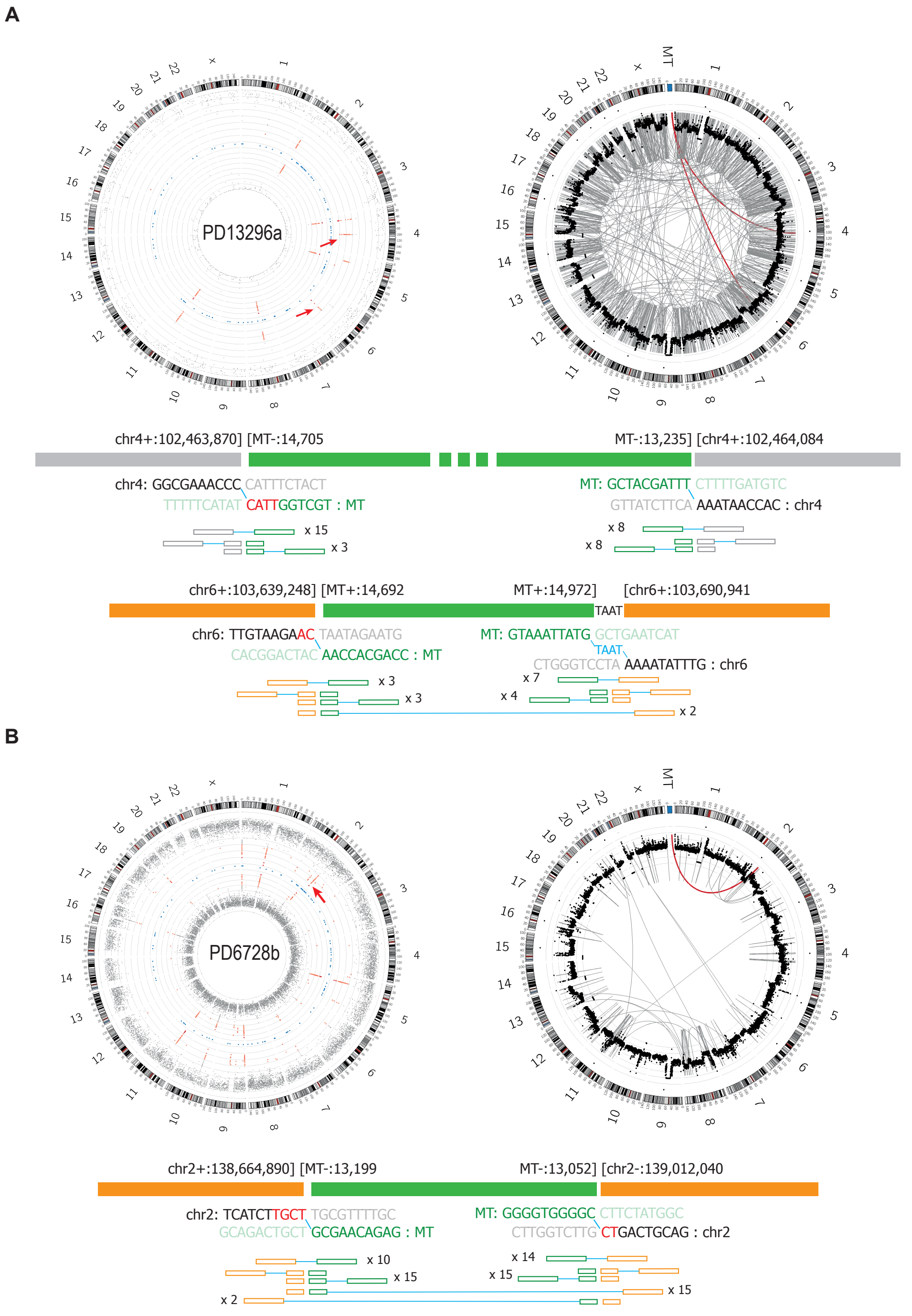

Supplemental Figure 3 | Genome and sequence context of mtDNA nuclear transfer events. Genome-wide discordant read clusters (first circos plot), chromosomal rearrangements (second circos plot), nucleotide-resolution mitochondrial-nuclear DNAbreakpoint junctions with microhomology (red letters) and number of supporting reads are graphically shown. **(A)** for PD13296a. **(B)** for PD6728b.
