# Supplementary material for: Frequent somatic transfer of mitochondrial DNA into the nuclear genome of human cancer cells
Source: Genome Res. 2015 Jun;25(6):814–24. doi: 10.1101/gr.190470.115 (PMC4448678; doi:10.1101/gr.190470.115)
Supplement: Supplemental Material [file supp_gr.190470.115_Supp_Figure7.pdf]

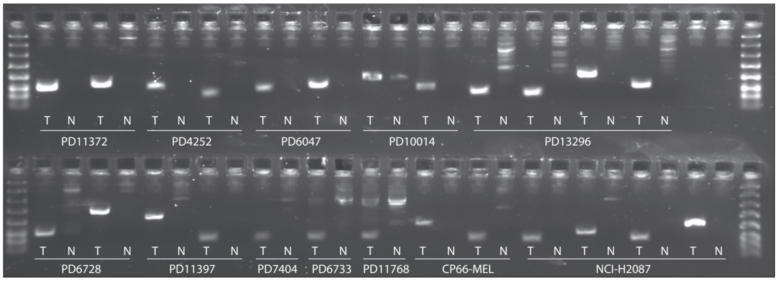

Supplemental Figure 7 | Agarose gel electrophoresis of breakpoint PCR validation .
These PCR products targeted 25 mtDNA-nuclear DNA fusion juctions. T: tumour sample, N: matched-normal sample. (According primer sequences are available in Supplemental Table 2).
