# Supplementary material for: Frequent somatic transfer of mitochondrial DNA into the nuclear genome of human cancer cells
Source: Genome Res. 2015 Jun;25(6):814–24. doi: 10.1101/gr.190470.115 (PMC4448678; doi:10.1101/gr.190470.115)
Supplement: Supplemental Material [file supp_gr.190470.115_Supp_Figure8.pdf]

A

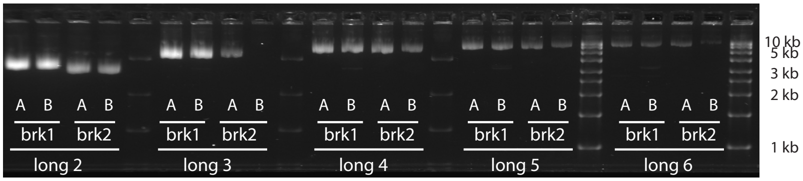

B

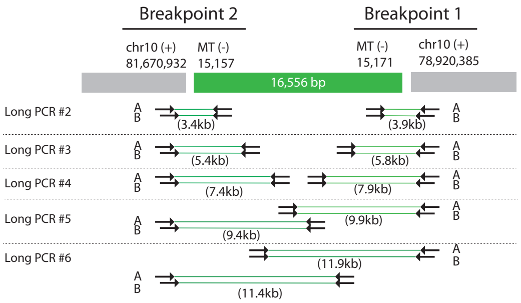

Supplemental Figure 8 | Agarose gel electrophoresis of long-PCR products. (A) These products confirm that almost entire mtDNA genome (16,556bp) was somatically transferred to the nuclear genome of PD11372a. Notation, product size and primer sequences for each PCR product are described in Supplemental Table 2 and **(B)** the schematic diagram.
