# Supplementary material for: Frequent somatic transfer of mitochondrial DNA into the nuclear genome of human cancer cells
Source: Genome Res. 2015 Jun;25(6):814–24. doi: 10.1101/gr.190470.115 (PMC4448678; doi:10.1101/gr.190470.115)
Supplement: Supplemental Material [file supp_gr.190470.115_Supp_Figure9.pdf]

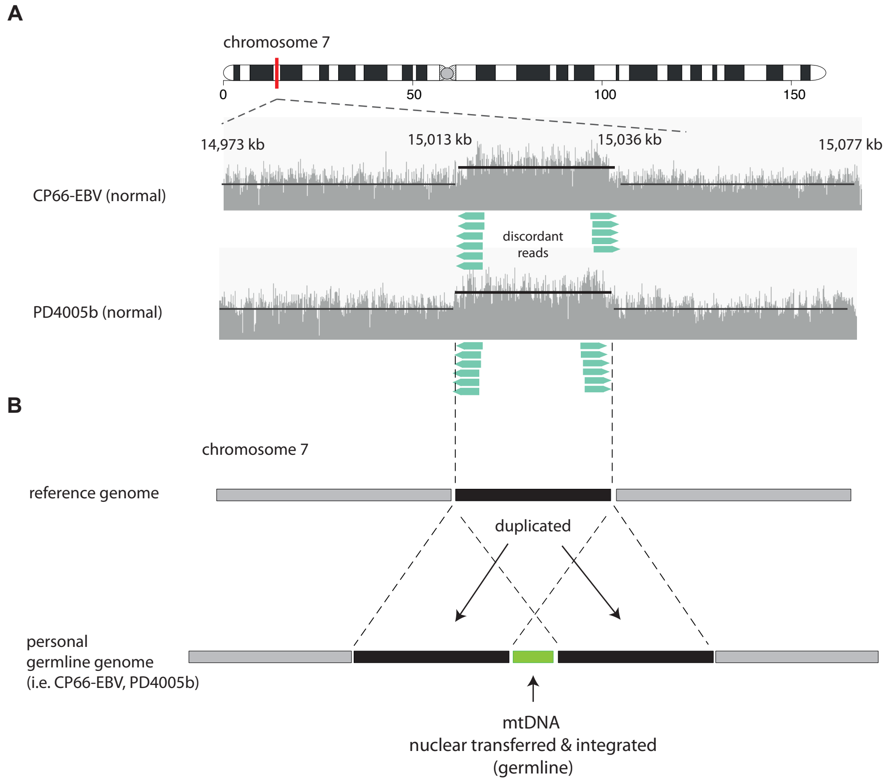

Supplemental Figure 9 | A novel germline mitochondrial-nuclear DNA fusion (Numt) combined with a local tandem duplication.
(A) Sequencing coverages from two unrelated normal samples are shown together with discordant read clusters. **(B)** Reconstructed personal genome sequences.
