# Supplementary material for: Frequent somatic transfer of mitochondrial DNA into the nuclear genome of human cancer cells
Source: Genome Res. 2015 Jun;25(6):814–24. doi: 10.1101/gr.190470.115 (PMC4448678; doi:10.1101/gr.190470.115)
Supplement: Supplemental Material [file supp_gr.190470.115_Supplemental_Material.docx]

**Supplemental Material**

Frequent somatic transfer of mitochondrial DNA into the nuclear genome of human cancer cells

Young Seok Ju, Jose M. C. Tubio, William Mifsud, Beiyuan Fu, Helen R. Davies, Manasa Ramakrishna, Yilong Li, Lucy Yates, Gunes Gundem, Patrick S. Tarpey, Sam Behjati, Elli Papaemmanuil, Sancha Martin, Anthony Fullam, Moritz Gerstung, ICGC Prostate Cancer Working Group, ICGC Bone Cancer Working Group, ICGC Breast Cancer Working Group, Jyoti Nangalia, Anthony R. Green, Carlos Caldas, Åke Borg, Andrew Tutt, Ming Ta Michael Lee, Laura J. van't Veer, Benita K. T. Tan, Sam Aparicio, Paul N. Span, John W.M. Martens, Stian Knappskog, Anne Vincent-Salomon, Anne-Lise Børresen-Dale, Jórunn Erla Eyfjörd, Adrienne M Flanagan, Christopher Foster, David E. Neal, Colin Cooper, Rosalind Eeles, Sunil R. Lakhani, Christine Desmedt, Gilles Thomas, Andrea L. Richardson, Colin A. Purdie, Alastair M. Thompson, Ultan McDermott, Fengtang Yang, Serena Nik-Zainal, Peter J. Campbell and Michael R. Stratton

**Contents**

**- Supplemental Text**

1. Novel germline mitochondrial sequence in the nuclear genome ----------------- 3
2. Frequent nuclear transfer of mtDNA in human cancer cells -------------------------- 3

**- Supplemental Tables**

1. Supp.Table.1: Sample and sequencing information ----------------------------------- 5
2. Supp.Table.3: Novel germline mtDNA sequences in the nucleus (Numts) -------- 5
3. Supp.Table.3: Primer sequences ----------------------------------------------------- 6
4. Association between mtDNA integration and transposable elements ---------------- 7

**- Ethical approval and the lists of ICGC working group participants**

1. Ethical Approval ----------------------------------------------------------------------- 8

2. ICGC Prostate cancer working group ----------------------------------------------------- 9

2. ICGC Breast cancer working group ----------------------------------------------------- 10

**Supplemental Text**

Novel germline mitochondrial sequence in the nuclear genome

In addition to the somatic transfers, we found 55 previously unreported mtDNA nuclear integrations that were shared between cancer and normal tissues from the same individual. 63.6% (n=35) of these integrations were found in only one individual and the population allele frequency for 98.2% of them (n=54) was 0.5% or less. Most are therefore likely to have taken place in the germline during recent human evolution (Supplemental Table 3). Almost all were simple insertions into the nuclear genome. However, one, which is shared by four samples, was combined with a tandem duplication of 22.3kb of nuclear DNA (Supplemental Fig. 9). The results indicate that nuclear transfer of mtDNA is ongoing in the human germline and, in some instances, is closely associated with intranuclear structural changes, similar to the observations reported here in somatic cells.

Frequent nuclear transfer of mtDNA in human cancer cells

We observe a much higher rate of mitochondrial DNA fusion to the nuclear genome in human somatic cells than in the human germline. With about 1000 cell divisions from the fertilized egg to the progenitor cell of the dominant cancer cell clone, the rate of somatic nuclear mtDNA fusion appears to be 2 x 10^-5^ event per cell per cell generation (pcpg), which is at least several hundreds times higher than the rate observed in the human germline (5 x 10^-8^ pcpg) across the evolution (Hazkani-Covo et al. 2010) (Methods).

Assuming that the intrinsic rates of double strand break generation and of ligation to a break in a different chromosome are similar for mitochondrial and nuclear DNA, our findings indicate that physical escape of mtDNA to make contact with nuclear DNA is frequent in some human somatic cells. Under conservative assumptions i) that >10% of mtDNA which escapes is integrated with nuclear DNA, and ii) that the rate of escape is constant during these cell divisions, the results suggest more than one mtDNA escape to the nucleus in 5,000 cell generations (2×10^-4^ pcpg). If the escape is a cancer cell specific phenomenon, its rate must be substantially higher. Our estimated escape frequency in human cells appears to be comparable to the rate observed in *Saccharomyces cerevisiae* (Thorsness et al. 1990)(2×10^-5^ pcpg) and to the rate of nuclear migration of chloroplast DNA in tobacco plants (~10^-4^ pcpg; Huang et al. 2003).

**Supplemental Tables**

**Supp. Table 1.** Sample and sequencing information of 559 primary and 28 cell-line tumor-normal pairs.

Ju_TableS1_SamplesInfo_withAccession.xlsx

Depicted below is a thumbnail of the file.

**
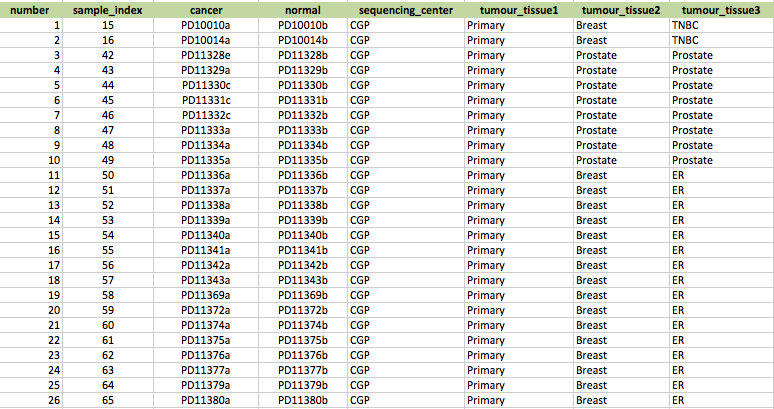
**

**Supp. Table 2.** List of novel germline mtDNA sequences in human nuclear genome.

Ju_TableS2_Inherited_mtDNA.xlsx

Depicted below is a thumbnail of the file.

**
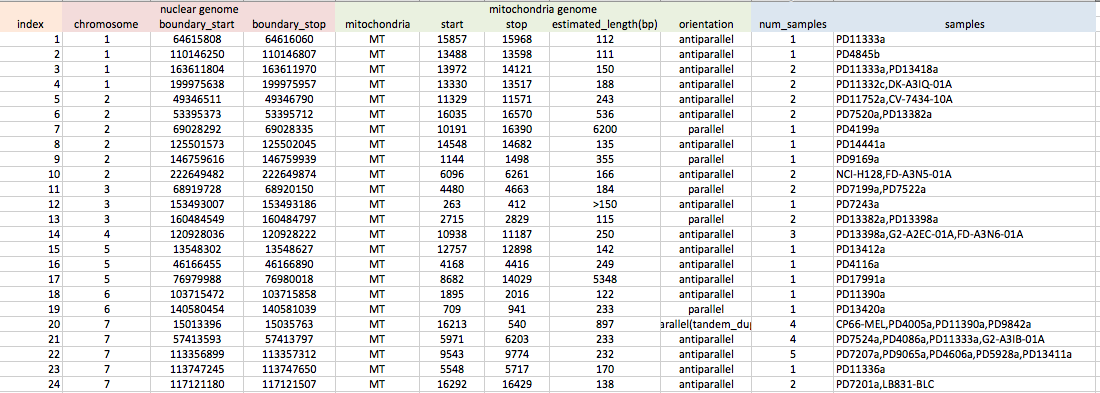
**

**Supp. Table 3.** Primer sequences for PCR validations.

Ju_TableS3_PrimerSequences.xlsx

Depicted below is a thumbnail of the file.


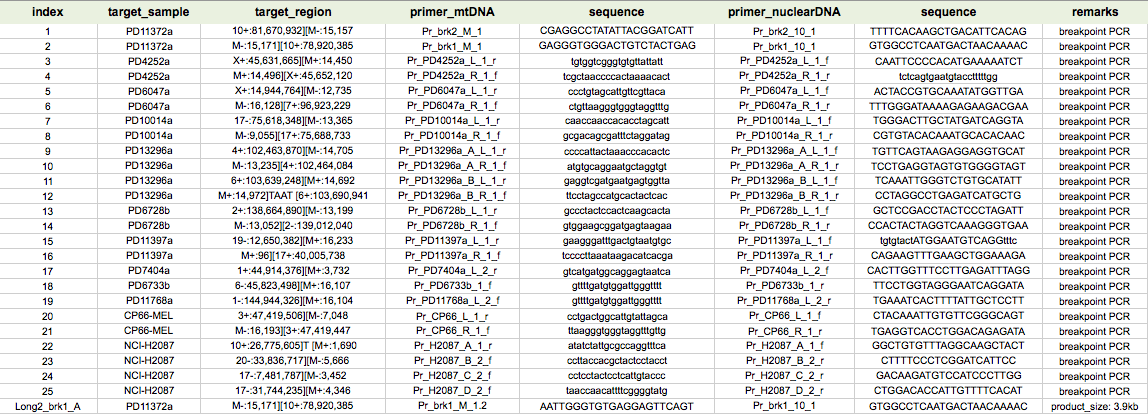


**Supp. Table 4.** Association between mtDNA integration and transposable elements.

Ju_TableS4_mtDNAinsertions_and_transposableelements.xlsx

Depicted below is a thumbnail of the file.

**
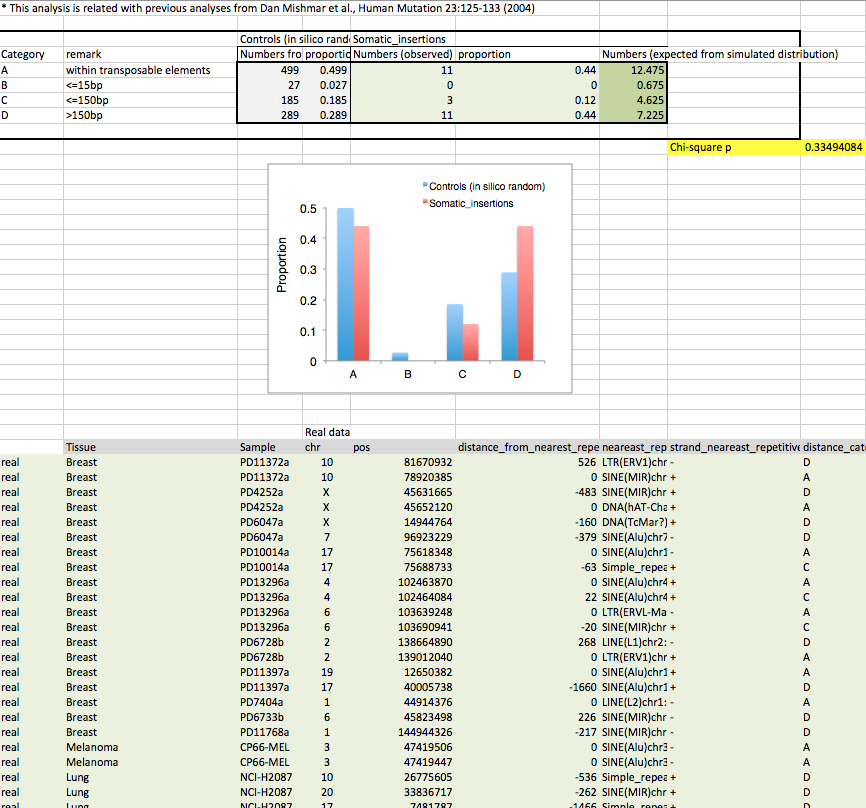
 Ethical approval references**

Genome Analysis of myeloid and lymphoid malignancies (10/H0306/40)

Genomic Analysis of Mesothelioma (11/EE/0444)

Myeloid and lymphoid cancer genome analysis (07/S1402/90)

The Treatment of Down Syndrome Children with Acute Myeloid Leukemia and Myelodysplastic Syndrome(AAML0431)

CLL (chronic lymphocytic leukaemia) genome analysis (07/Q0104/3)

Cancer Genome Project - Global approaches to characterising the molecular basis of paediatric ependymoma

(05/MRE04/70)

PREDICT-Cohort (09/H0801/96)

ICGC Prostate (Evaluation of biomarkers in urological diseases) (LREC 03/018)

ICGC Prostate (779) (Prostate Complex CRUK Sample Cohort) (MREC/01/4/061)

ICGC Prostate (Tissue collection at radical prostatectomy) (CRE-2011.373)

Somatic molecular genetics of human cancers, melanoma and myeloma (Dana Farber Cancer Institute)(08/H0308/303)

Breast Cancer Genome Analysis for the International Cancer Genome Consortium Working Group (09/H0306/36)

Genome analysis of tumours of the bone (09/H0308/165)

**Participants in ICGC prostate cancer working group**

Colin Cooper^1,2,16^, Rosalind Eeles^1,3,16^, David Wedge^4^, Peter Van Loo^4,5^, Gunes Gundem^4^, Ludmil Alexandrov^4^, Barbara Kremeyer^4^, Adam Butler^4^, Andrew Lynch^6^, Sandra Edwards^1^, Niedzica Camacho^1^, Charlie Massie^7^, ZSofia Kote-Jarai^1^, Nening Dennis^3^, Sue Merson^1^, Jorge Zamora^4^, Jonathan Kay^7^, Cathy Corbishley^8^, Sarah Thomas^3^, Serena Nik-Zainai^4^, Sarah O’Meara^4^, Lucy Matthews^1^, Jeremy Clark^2^, Rachel Hurst^2^, Richard Mithen^9^, Susanna Cooke^4^, Keiran Raine^4^, David Jones^4^, Andrew Menzies^4^, Lucy Stebbings^4^, Jon Hinton^4^, Jon Teague^4^, Stuart McLaren^4^, Laura Mudie^4^, Claire Hardy^4^, Elizabeth Anderson^4^, Olivia Joseph^4^, Victoria Goody^4^, Ben Robinson^4^, Mark Maddison^4^, Stephen Gamble^4^, Christopher Greenman^10^, Dan Berney^11^, Steven Hazell^3^, Naomi Livni^3^, Cyril Fisher^3^, Christopher Ogden^3^, Pardeep Kumar^3^, Alan Thompson^3^, Christopher Woodhouse^3^, David Nicol^3^, Erik Mayer^3^, Tim Dudderidge^3^, Nimish Shah^7^, Vincent Gnanapragasam^7^, Peter Campbell^4^, Andrew Futreal^4,16^, Douglas Easton^12,16^, Anne Y Warren^13^, Christopher Foster^14,16^, Michael Stratton^4,16^, Hayley Whitaker^7^, Ultan McDermott^4,16^, Daniel Brewer^1,2^, David Neal^7,15,16^.

**Affiliations**

^1^Division of Genetics and Epidemiology, The Institute Of Cancer Research, Sutton, UK

^2^Department of Biological Sciences and School of Medicine, University of East Anglia, Norwich, UK

^3^Royal Marsden NHS Foundation Trust, London and Sutton, UK

^4^Cancer Genome Project, Wellcome Trust Sanger Institute, Hinxton, UK

^5^Human Genome Laboratory, Department of Human Genetics, VIB and KU Leuven, Leuven, Belgium

^6^Statistics and Computational Biology Laboratory, Cancer Research UK Cambridge Research Institute, Cambridge, UK

^7^Urological Research Laboratory, Cancer Research UK Cambridge Research Institute, Cambridge, UK

^8^Department of Histopathology, St Georges Hospital, London, UK

^9^Institute of Food Research, Norwich Research Park, Norwich, UK

^10^School of Computing Sciences, University of East Anglia, Norwich, UK

^11^Department of Molecular Oncology, Barts Cancer Centre, Barts and the London School of Medicine and Dentistry, London, UK

^12^Centre for Cancer Genetic Epidemiology, Department of Oncology, University of Cambridge, Cambridge, UK

^13^Department of Histopathology, Cambridge University Hospitals NHS Foundation Trust, Cambridge, UK

^14^Bostwick Laboratories, London, UK

^15^Department of Surgical Oncology, University of Cambridge, Addenbrooke's Hospital, Cambridge, UK

^16^Senior Principal Investigators of the Cancer Research UK funded ICGC Prostate Cancer Project

**Participants in ICGC breast cancer working group**

Elena Provenzano^1^, Marc van de Vijver^2^, Andrea L Richardson^3,4^, Colin Purdie^5^, Sarah Pinder^6^, Gaetan MacGrogan^7^, Anne Vincent-Salomon^8,9^, Denis Larsimont^10^, Dorthe Grabau^11^, Torill Sauer^12^, Øystein Garred^12^, Anna Ehinger^13^, Gert G Van den Eynden^14^, C.H.M. van Deurzen^15^, Roberto Salgado^29^, Jane E Brock^4^, Sunil R Lakhani^16,17,18^, Dilip D Giri^19^, Laurent Arnould^20^, Jocelyne Jacquemier^21^, Isabelle Treilleux^22^, Carlos Caldas^1,23^, Suet-Feung Chin^23^, Aquila Fatima^3^, Alastair M Thompson^24^, Alasdair Stenhouse^24^, John Foekens^25^, John Martens^25^, Anieta Sieuwerts^25^, Arjen Brinkman^26^, Henk Stunnenberg^26^, Paul N. Span^27^, Fred Sweep^28^, Christine Desmedt^29^, Christos Sotiriou^29^, Gilles Thomas^30^, Annegein Broeks^31^, Anita Langerod^32^, Samuel Aparicio^33^, Peter Simpson^18^, Laura van 't Veer^34,35^, Jórunn Erla Eyfjörd^36^, Holmfridur Hilmarsdottir^36^, Jon G Jonasson^37,38^, Anne-Lise Børresen-Dale^32,39^, Ming Ta Michael Lee^40^, Bernice Huimin Wong^41^, Benita Kiat Tee Tan^42^, Gerrit K.J. Hooijer^2^, Andrew Tutt^43^

**Affiliations**

^1^Cambridge Breast Unit, Addenbrooke’s Hospital, Cambridge University Hospital NHS Foundation Trust and NIHR Cambridge Biomedical Research Centre, Cambridge CB2 2QQ, UK

^2^Department of Pathology, Academic Medical Center, Meibergdreef 9, 1105 AZ Amsterdam, The Netherlands

^3^Department of Cancer Biology, Dana-Farber Cancer Institute, 450 Brookline Ave., Boston, Massachusetts 02215, USA

^4^Department of Pathology, Brigham and Women's Hospital, Harvard Medical School, 75 Francis St., Boston, Massachusetts 02115, USA.

^5^East of Scotland Breast Service, Ninewells Hospital, Dundee, United Kingdom

^6^Department of Research Oncology, Guy’s Hospital, King’s Health Partners AHSC, King’s College London School of Medicine, London SE1 9RT, UK

^7^Institut Bergonié, 229 cours de l’Argone, 33076, Bordeaux, France

^8^Institut Curie, Department of Tumor Biology, 26 rue d’Ulm, 75248 Paris cédex 05, France.

^9^Institut Curie, INSERM Unit 830, 26 rue d’Ulm, 75248 Paris cédex 05, France

^10^Department of Pathology, Jules Bordet Institute, Brussels 1000, Belgium

^11^Department of Pathology, Skåne University Hospital, Lund University, SE-221 85 Lund, Sweden

^12^Department of Pathology, Oslo University Hospital Ulleval and University of Oslo, Faculty of Medicine and Institute of Clinical Medicine, Oslo, Norway.

^13^Department of Gynecology & Obstetrics, Department of Clinical Sciences, Lund University, Skåne University Hospital Lund, SE-221 85 Lund, Sweden

^14^Translational Cancer Research Unit, GZA Hospitals St.-Augustinus, Antwerp, Belgium.

^15^Department of Pathology, Erasmus Medical Center, Rotterdam, the Netherlands.

^16^The University of Queensland, School of Medicine, Herston, Brisbane, QLD 4006, Australia

^17^Pathology Queensland: The Royal Brisbane & Women’s Hospital, Brisbane, QLD 4029, Australia

^18^The University of Queensland, UQ Centre for Clinical Research, Herston, Brisbane, QLD 4029, Australia

^19^Department of Pathology, Memorial Sloan-Kettering Cancer Center, New York, NY, USA

^20^Centre Georges-François Leclerc, 1 rue du Professeur Marion, 21079, Dijon, France

^21^Institut Paoli Calmettes, biopathology department, 232 Bd Ste Marguerite, 13009, Marseille, France

^22^Centre Léon Bérard, Lyon, France; Université Claude Bernard Lyon1 - Université de Lyon, Lyon, France.

^23^Department of Oncology, University of Cambridge and Cancer Research UK Cambridge Research Institute, Li Ka Shin Centre, Cambridge CB2 0RE

^24^Department of Surgical Oncology, University of Texas MD Anderson Cancer Center, Houston, Texas, USA.

^25^Erasmus MC Cancer Institute, Erasmus University Medical Center, Rotterdam, The Netherlands.

^26^Radboud University, Department of Molecular Biology, Faculty of Science, Nijmegen Centre for Molecular Life Sciences, 6500 HB Nijmegen, The Netherlands.

^27^Department of Radiation Oncology, Radboud University Medical Centre, Nijmegen, The Netherlands

^28^Department of Laboratory Medicine, Radboud University Medical Centre, Nijmegen, The Netherlands

^29^Breast Cancer Translational Research Laboratory, Institut Jules Bordet, Université Libre de Bruxelles, Brussels, Belgium

^30^Universite Lyon1, INCa-Synergie, Centre Leon Berard, 28 rue Laennec Lyon Cedex 08 France

^31^Department Experimental Therapy, The Netherlands Cancer Institute, Plesmanlaan 121, 1066 CX Amsterdam, The Netherlands

^32^Department of Genetics, Institute for Cancer Research, The Norwegian Radium Hospital, Oslo University Hospital, O310 Oslo, Norway

^33^Department of Molecular Oncology, BC Cancer Agency, 675 W10th Avenue, Vancouver V5Z 1L3

^34^The Netherlands Cancer Institute, Division of Molecular Carcinogenesis, Amsterdam, The Netherlands.

^35^Department of Surgery, University of California, San Francisco, San Francisco, California, United States of America.

^36^Cancer Research Laboratory, Faculty of Medicine, University of Iceland, Reykjavik, Iceland

^37^Department of Pathology, University Hospital, Reykjavik, Iceland

^38^Icelandic Cancer Registry, Icelandic Cancer Society, Skogarhlid 8, P.O.Box 5420, 125, Reykjavik, Iceland

^39^ The K.G. Jebsen Center for Breast Cancer Research, Institute for Clinical Medicine, Faculty of Medicine, University of Oslo, Norway

^40^Laboratory for International Alliance on Genomic Research, RIKEN Center for Integrative Medical Sciences, Yokohama, Japan

^41^NCCS-VARI Translational Research Laboratory, National Cancer Centre Singapore, 11 Hospital Drive, 169610, Singapore

^42^Department of General Surgery, Singapore General Hospital, Singapore

^43^ Breakthrough Breast Cancer Research Unit, Research Oncology, King’s College London, Guy’s Hospital, London SE1 9RT, UK.
